# AI-Driven Analysis of Wrist-Worn Sensor Data for Monitoring Individual Treatment Response and Optimizing Levodopa Dosing in Parkinson’s Disease

**DOI:** 10.3390/s25237273

**Published:** 2025-11-28

**Authors:** Mathias Sander, Moritz R. Messner, Sina K. Knapp, Franz M. J. Pfister, Urban M. Fietzek

**Affiliations:** 1 Orbit Health GmbH, 81667 Munich, Germany; mathias@orbit.health (M.S.);; 2 Department of Neurology, University Hospital, Ludwig-Maximilians-University (LMU), 80539 Munich, Germany; moritz.messner@campus.lmu.de; 3 Department of Neurology and Clinical Neurophysiology, Schön Klinik München Schwabing, 80804 Munich, Germany

**Keywords:** Parkinson’s disease, wearable sensors, motor fluctuation, continuous monitoring, levodopa, levodopa cycle, symptom severity

## Abstract

Parkinson’s Disease is a progressive neurodegenerative disorder marked by motor fluctuations in later disease stages that complicate treatment with levodopa. Traditional approaches to dosing often fail to capture the complex and dynamic nature of these fluctuations. In this study, we present the PD9™ algorithm, a novel approach to continuous motor state monitoring using data from a wrist-worn inertial measurement unit sensor. The algorithm provides minute-by-minute assessments of motor state severity on a unified scale quantifying bradykinesia, dyskinesia, and ON states. Data collected from 67 patients over 55,482 min were analyzed to assess levodopa response cycles. Across 218 identified levodopa cycles, the algorithm revealed reproducible patterns of symptom development based on the motor state at the time of levodopa administration. In particular, levodopa doses administered during non-ideal motor states (e.g., during dyskinesia) highlighted the limitations of fixed, empirically determined dosing regimens and underscore the need for individualized therapy, based on motor state. These findings demonstrate how AI-enabled continuous monitoring could help realize a more personalized treatment of Parkinson’s disease and improve patient outcomes.

## 1. Introduction

Parkinson’s Disease (PD) is the fastest-growing and the second most common neurodegenerative disease after Alzheimer’s Disease. An estimated number of 10 million people suffered globally from PD in 2020 [1,2]. By 2040, likely more than 17 million people will live with PD [3,4]. PD is a progressive neurodegenerative disease presenting motor and non-motor symptoms [5,6], and its prevalence increases with age and has both genetic as well as environmental underlying causes [7,8,9].

The main line of treatment for PD currently involves levodopa administration to relieve symptoms, mainly slowness of movement and loss of movement spontaneity. In the early stages of PD, levodopa (LD) therapy is highly effective in alleviating symptoms. However, as the disease progresses, fluctuations in medication efficacy—commonly termed “on–off” fluctuations—become more pronounced, especially in response to LD. These fluctuations result in alternating phases of symptom relief and exacerbation. During “on” phases, the medication effectively improves motor function, allowing relatively normal mobility. In contrast, “off” phases are characterized by significant motor impairments, including muscle stiffness, rigidity and bradykinesia. Additionally, on the other side of the movement spectrum, levodopa dosing may cause excessive response, leading to involuntary hyper-mobility, known as dyskinesia [7,10,11].

Motor fluctuations arise due to the brain’s declining capacity to process and store dopamine uniformly as PD advances, complicating consistent dopamine delivery with oral medications like LD. With each dose of LD, a new “levodopa cycle” is initiated: absorption in the small intestine occurs rapidly, and the peak plasma concentration is typically reached within 0.5–2 h after administration. After passing the blood–brain barrier, LD is metabolized to dopamine, activating postsynaptic dopaminergic receptors. After reaching peak plasma concentration, LD plasma concentrations begin to decline, resulting in significantly lower levels around 4–5 h post-administration [12,13]. Pharmacokinetics of orally administered LD are likely to cause motor fluctuations: when peak LD blood concentration is reached, patients might experience a dyskinetic (DYS) motor state, whereas a decrease in LD blood concentration might result in an OFF state [14,15,16]. Factors contributing to motor fluctuations include irregular gastrointestinal absorption, often slowed in patients with PD (PwPD), and diminished dopamine storage, which accelerates the reduction in dopamine levels in the central nervous system (CNS). Consequently, these fluctuations compromise patients’ quality of life by introducing unpredictability and diminishing control over bodily movements [7,10,11].

The primary goal of Parkinson’s disease (PD) treatment is to minimize OFF periods and dyskinesias (DYS) while maintaining patients in an optimal, symptom-free motor state for as long as possible [17,18]. To achieve this, therapeutic strategies aim to provide a more continuous dopaminergic stimulation by reducing the pulsatility of LD administration [19,20]. This can be accomplished through fractionated dosing, in which smaller doses are administered at shorter intervals to smooth plasma concentration profiles and mitigate motor fluctuations. Adjunctive use of enzyme inhibitors, such as catechol-O-methyltransferase (COMT) and monoamine oxidase B (MAO-B) inhibitors, further prolongs LD’s half-life and enhances its bioavailability, thereby supporting more stable dopamine levels in the brain [21,22,23].

However, conventional fixed-dose or standard-interval regimens, though convenient in practice, often fail to sustain stable dopaminergic stimulation throughout the day. As a result, individualized medication scheduling has become an essential aspect of modern PD management [18,20,24]. Determining the optimal dose and timing for each patient remains challenging due to the complex interplay of factors influencing LD pharmacokinetics and pharmacodynamics, including variability in gastrointestinal absorption, dietary effects, circadian rhythms, and patient arousal state [24].

In Germany, the current standard of care for PwPD consists of structured but brief clinical visits every 3–12 months, during which treatment regimens are adjusted according to disease progression and individual needs. However, capturing a comprehensive picture of a patient’s symptom profile during such limited consultations is difficult, given the dynamic and individualized nature of PD. Therefore, real-world, high-frequency monitoring of symptom dynamics in relation to medication intake is increasingly recognized as critical for optimizing treatment schedules and outcomes. In line with this, current clinical guidelines recommend regular monitoring of both motor and non-motor symptoms to facilitate personalized therapy adjustments [25,26,27,28].

Wearable sensor technologies, combined with artificial intelligence (AI) algorithms that translate raw data into clinically relevant insights on PD symptoms, hold significant promise in this context. While numerous algorithms have recently been developed to explore this potential [27,28,29,30,31,32], there are already systems on the market that are approved as medical devices and suitable for routine clinical use [27,33,34].

To address the need for continuous monitoring of PwPD, we developed a high-resolution motor state assessment algorithm using data collected from a wrist-worn IMU sensor which is transformed by a fully convolutional neural network [35,36]. Algorithmically transformed data are shown as a continuous motor state graph in a symptom severity scale called PD9^TM^ including information about the motor state (dyskinesia, ON, OFF/bradykinesia; and their severity). PD9^TM^ is based on a combination of the Movement Disorder Society-Unified Parkinson Disease Severity Scale (MDS-UPDRS) [37] global bradykinesia item and the modified Abnormal Involuntary Movement Scale (mAIMS) upper limb dyskinesia item [38]. The continuous and long-term monitoring of the motor state gives insights into the varying motor states of PwPD per minute throughout the day, which is a prerequisite, to enable optimized treatment adjustment [39].

The aim of this study is to understand if our continuous monitoring and machine learning techniques can capture motor fluctuations of PwPD treated with LD and identify ways of improving treatment adjustments.

## 2. Materials and Methods

### 2.1. Ethical Vote and Patient Consent

The ethical board of the Technical University of Munich, Germany (TUM) approved this clinical investigation (No. 234/16S) on 30 June 2016. With their written consent, all patients included in the study agreed to the recording and analysis of their anonymized data. The authors confirm that all experiments were performed in accordance with relevant guidelines and regulations.

### 2.2. Study Cohort

The entire available patient cohort consisted of 95 patients diagnosed with PD according to UK Brain Bank criteria [35,36]. There were 60,712 min of algorithmic motor symptom data available.

Exclusion Criteria:

Patients were excluded from all analyses if a complete LD dose cycle could not be derived from the available algorithmic motor state data. A complete LD cycle was defined as the presence of algorithmic motor state data covering the period from 20 min before to 90 min after a scheduled medication intake.

Hence, 67 patients with at least one full LD cycle were included, leading to a study cohort with 55,482 min of algorithmic motor symptom data.

### 2.3. Algorithm

The PD9^TM^ algorithm is a proprietary algorithm which is part of the software as a medical device (SaMD) called Neptune™ (Orbit Health GmbH, Munich, Germany). Neptune is a CE-marked medical device under the current European medical device regulation (Regulation (EU) 2017/745). Its algorithm enables continuous monitoring of motor symptoms and treatment responses of PwPD. This continuous information is intended to support healthcare professionals in treatment adjustment and personalization.

**Figure 1:**
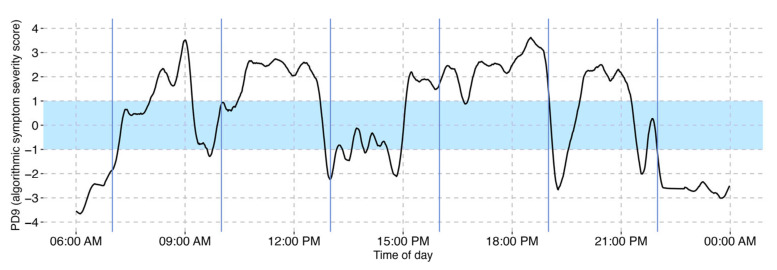
The PD9^TM^ graph depicts the PD motor state on a range from −4 to +4 over the course of 24 h. Values from −1 to −4 represent increasing severity of bradykinesia, and +1 to +4 represents increasing severity of dyskinesia. The area between −1 and +1 corresponds to the ON state. Vertical solid blue lines represent the scheduled time of medication intake. The dashed lines represent grid lines that are included as a guide to the eye.

The PD9^TM^ algorithm is a deep learning model that converts raw accelerometer and gyroscope data from an IMU sensor into minute-by-minute values on a scale known as PD9^TM^. This scale continuously measures Parkinson’s symptom severity, ranging from −4 to +4. Values from −1 to −4 represent increasing levels of bradykinesia/OFF states, while values from +1 to +4 indicate increasing severity of dyskinesia. The ON state is represented by the range from −1 to +1 (excluding +1 and −1). By integrating bradykinesia/OFF, ON, and dyskinesia into a single scale, PD9^TM^ enables comprehensive visualization of motor states, detection of subtle changes in motor function, and identification of motor fluctuations (see Figure 1).

The algorithm is built on a fully convolutional network (FCN) architecture, trained using data collected from individuals with Parkinson’s disease who wore a smartwatch during daily activities. Simultaneously, a movement disorder expert observed the patients in both free-living and hospital environments, rating their motor symptoms every minute using items from validated scales, namely MDS-UPDRS item III.14 and item 5 of mAIMS. This experimental setup [35] generated a comprehensive, clinically labeled dataset, enabling the supervised training of the FCN model and ultimately resulting in the development of the PD9^TM^ algorithm. Detailed descriptions of the algorithm, training, and validation have been published previously [36].

### 2.4. Statistics

- Levodopa cycles

To assess single LD cycles, algorithmic motor symptom data windows starting from 20 min before time of medication (t = 0) intake until 90 min after medication intake were extracted. This allowed the assessment of the motor state at time of medication intake and the motor symptom response to medication intake.

For this analysis, only complete data windows were utilized.

- Motor state at time of medication intake

From the LD cycles, the motor states (ON, OFF, DYS) at time of medication intake were deduced using the value of the PD9^TM^ at t = 0 as follows:

Motor state is classified as

- OFF if PD9^TM^ ≤ −1
- ON if −1 < PD9^TM^ < +1
- DYS if PD9^TM^ ≥ 1

Motor symptom response to medication was analyzed using descriptive statistics, including boxplots, as well as the mean and standard error of the mean at 5-min intervals after medication intake. The analysis was conducted across all medication cycles combined and separately based on the patients’ motor state at the time of intake.

## 3. Results

### 3.1. Study Population and Data Collected

Data from 67 patients (30 females and 37 males) included in the clinical study are represented here with clinical characteristics described in Table 1.

A total of 55,482 min of algorithm-transformed motor state data was analyzed from patients having at least one full LD cycle. On average, 828 ± 1364 min of data (minimum: 152 min, maximum: 5907 min) and 3.3 ± 5.9 LD cycles (minimum: 1, maximum: 25) in 1.4 ± 1.2 recording sessions (minimum: 1, maximum: 6) per patient were collected. A recording session refers to one day of data recording with at least one full LD cycle covered.

### 3.2. Visualization of Motor Symptom Severity

**Figure 2:**
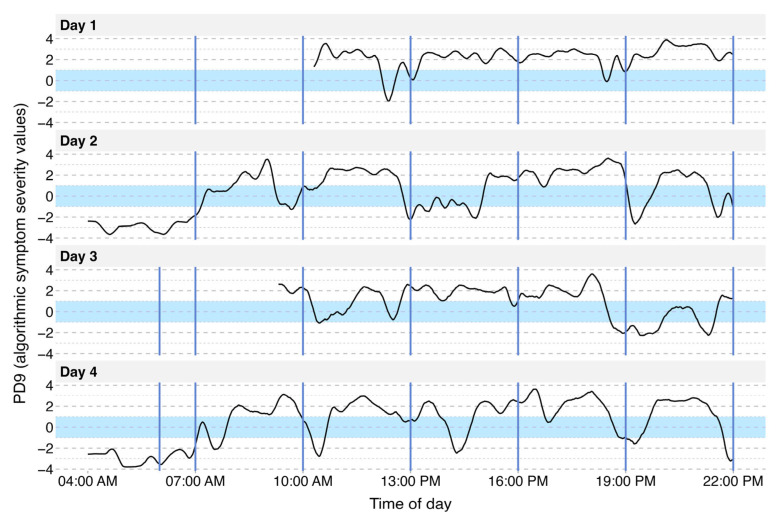
Visualization of the fluctuating motor state on four continuous days. Example graph of a 64-year-old female patient. Data are represented on the PD9^TM^ scale from −4 to +4 with −1 to −4 capturing increasing severity of bradykinesia/OFF and +1 to +4 capturing increasing severity of dyskinesia. The range of −1 to +1 (excluding +1 and −1) marks the ON state. Solid blue lines represent the prescribed LD intake time. During days 1 and 2, the prescribed treatment included five immediate-release levodopa (LD) doses administered at three-hour intervals between 7:00 a.m. and 7:00 p.m. and one controlled-release LD dose at 10:00 p.m. On days 3 and 4, the regimen was adjusted by adding a controlled-release LD dose at 6:00 a.m. On days 1 and 3, nighttime and morning data are missing. Full-day recordings were not required by the study protocol and hence patients were allowed to take off the wrist sensor during the night.

Figure 2 provides insights from four continuous days of motor state data collected from a 64-year-old female patient diagnosed with PD 12 years prior. The patient presented symptoms including morning akinesia, sudden ON/OFF fluctuations, and wearing-off episodes. The graph displays the PD9^TM^ rating for each minute on a continuous scale from −4 to +4 (detailed in Materials and Methods). In this example, daily curves illustrate motor state fluctuations over the course of each day. After the initial morning medication intake (indicated by dashed lines), the patient transitions from an OFF state (characterized by morning akinesia) to an ON state, eventually reaching a dyskinetic phase. Throughout the day, abrupt motor state shifts from dyskinesia back to the OFF state are evident, notably around 10:00 and 19:00. This motor state graph effectively reflects the morning akinesia, as well as wearing-off and sudden ON/OFF fluctuations, which is also in agreement with what is observed and reported by the treating physician.

### 3.3. Identification of Single L-DOPA Cycles

**Figure 3:**
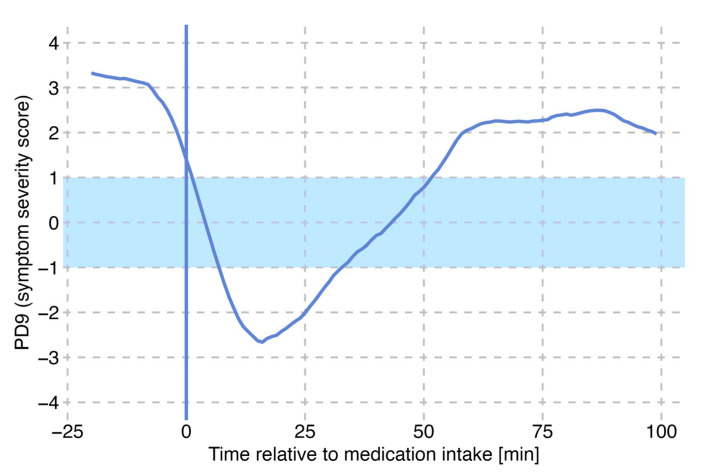
Visualization of orally administered LD cycle. The solid blue line (t = 0) indicates the time of LD intake. The blue area indicates the symptom-free ON-range.

In the daily motor state patterns of the patient shown in Figure 2, an observable effect of LD intake on motor state is evident. A magnified view of a single medication effect on Day 2 is presented in Figure 3. After experiencing a wearing-off period, the patient takes a new dose of Levodopa. Initially, the motor state curve shows a continuous decline into the OFF state followed by a shift towards the ON state approximately 20 min post-medication, reflecting the onset of the LD action. The motor state then stabilizes in a mildly dyskinetic state, further illustrating the temporal effects of LD on motor state regulation.

218 L-DOPA cycles are extracted and analyzed from the available motor state and medication intake times data as described in Materials and Methods. Tracking the motor states at the time of Levodopa intake for all patients in the study revealed an intriguing finding: Levodopa was administered predominantly in the OFF state but also during the ON and dyskinetic states. Although the majority of Levodopa doses were administered during the OFF state, nearly a quarter of administrations occurred during the dyskinetic state (see Table 2).

### 3.4. Motor States as an Effect of Levodopa Cycle

To assess how motor state at the time of LD administration influences subsequent motor response, we analyzed symptom severity scores at 5 min intervals over a 100 min post-administration period.

**Figure 4:**
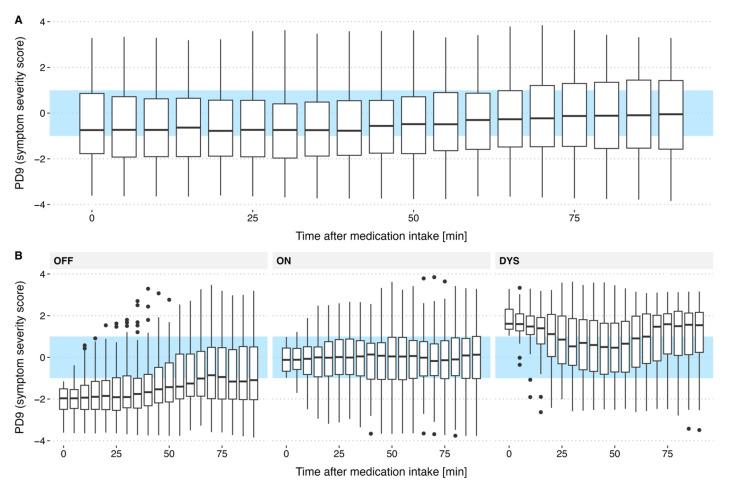
Change in motor symptoms following medication across all levodopa (LD) cycles recorded from the 67 patients. Algorithmic motor state data were analyzed only every five minutes to improve visual clarity compared to displaying one boxplot per minute. The blue regions in the graphs indicate the symptom-free ON-range. (**A**) Analysis of all 218 L-DOPA cycles shows overall stable motor symptom severity with a limited average effect of medication. (**B**) Stratifying by motor state at the time of medication intake reveals distinct response patterns visualized by the change in median: patients in the OFF state exhibit reduced OFF severity (increase median PD9 from −2 to −0.7); those in the ON state show minimal average changes (median remains almost constant); and patients in the dyskinetic state experience a temporary reduction in dyskinesia before returning to higher severity levels (change in median PD9 from 1.5 to 0.5 and back to 1.5). The wide variability in responses depicted by the width of the boxplots underscores significant inter- and intra-individual differences in medication effects. The observed variability may in part be attributable to confounding factors, including diet and stress, which were not controlled for in the present study.

Our findings (Figure 4) revealed distinct response patterns based on the initial motor state. When Levodopa was taken in the ON state, motor symptom severity remained relatively stable. In contrast, doses taken during the dyskinetic state were marked by an initial symptom reduction over 40–50 min (from ~1.5 to 0.75), followed by a return to baseline dyskinetic severity.

The most pronounced changes were observed when LD was administered in the OFF state, with symptom severity decreasing from −2 to −1 over 70 min.

**Figure 5:**
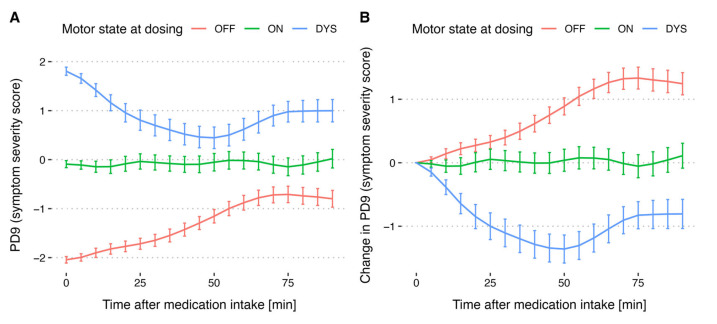
(**A**) Trend in algorithm-derived symptom severity scores over medication cycles for patients where medication was given in the ON-state, OFF-state and dyskinesia-state. (**B**) Changes from baseline (PD9(t) − PD9 (t = 0)) in algorithm-predicted symptom severity scores over medication cycles for patients who received LD in the different motor states. Graphs show mean ± standard error for *n* = 218 datasets: ON-state (n = 68), OFF-state (n = 99) and dyskinesia-state (n = 51). mean ± standard error is calculated every five minutes to enhance visual clarity compared to minute-by-minute depiction. The visualization clearly illustrates the behavior discussed in Figure 4. For patients taking medication in the OFF state, a clear symptom relief can be observed. In patients taking medication in the DYS state, an initial decrease in dyskinesia severity is followed by an increase as the medication takes effect. Medications administered in the ON state help patients remain stable and largely symptom-free.

Calculating mean and standard error of PD9 values every five minutes (Figure 5A) allows for a more comprehensive visualization of the medication effect on symptom severity. When the medication is taken during the ON state, average symptom severity shows no noticeable change. If taken during the OFF state, symptom severity is decreased which is highlighted by an increase in the average PD9 rating from −2 to −0.75. If taken during the DYS state, PD9 rating decreases highlighting an initial decrease in dyskinesia severity followed by an increase in dyskinesia severity after about 50 min. Normalization of the curves by subtracting the PD9 value at medication intake time reveals that LD intake during the OFF state has the greatest impact on reducing symptom severity (Figure 5B).

## 4. Discussion

The presented results provide evidence supporting the use of AI-powered continuous motor symptom monitoring via wearable sensors to individualize LD therapy in PD. Using the PD9™ algorithm, which processes inertial data from a wrist-worn sensor, we analyzed 218 LD cycles across 67 patients and demonstrated that individual treatment responses can be monitored effectively.

LD intake during the OFF state resulted in the most significant symptom improvement as expected. However, this benefit also implies that the medication was taken too late, after motor deterioration had already occurred. As the clinical goal is to prevent OFF states and maintain stable motor function in the ON state, such patterns indicate suboptimal medication timing. Conversely, LD intake during the ON state led to minimal change in symptom severity, suggesting successful symptom control. However, nearly one-quarter of LD doses were taken during dyskinetic states, raising concern for potential overdosing or overly aggressive titration. These findings underscore the importance of individual, motor-state-informed dosing and highlight the limitations of fixed, empirically set schedules [18,24,40,41].

Our results are consistent with a growing body of research demonstrating the clinical potential of wearable sensor systems for PD monitoring. Devices such as the Personal KinetiGraph (PKG^®^), STAT-ON™, and PDMonitor^®^ provide continuous, objective symptom tracking, enabling identification of OFF periods, dyskinesias, and treatment response patterns [42,43,44,45]. PKG use in routine care has led to treatment changes in up to one-third of clinic visits and improved symptom control [44]. STAT-ON has shown ON/OFF state classification sensitivity and specificity exceeding 90%, outperforming subjective diaries in sensitivity and agreement with clinical assessments [45]. These findings support the clinical impact of wearable-guided symptom awareness and treatment adjustments.

Advances in AI have improved the interpretation of wearable raw motion data significantly. Machine learning models such as support vector machines, convolutional neural networks, and other deep learning approaches have been applied to enable classification of PD motor states and predict treatment responses [27,28,29,30,31,32,33,34,35,36]. Studies using AI-driven scales have shown strong correlation with clinical assessments and enabled real-time monitoring of therapeutic windows [42].

Importantly, several proof-of-concept systems have demonstrated the feasibility of algorithmic LD dose [17,46] or DBS settings [47] optimization. Sensor-based dosing systems have used wearable data and pharmacokinetic modeling to simulate and recommend personalized schedules, achieving close agreement with expert clinician decisions [46]. Reinforcement learning approaches have also been applied to develop optimized medication regimens [48,49]. While still emerging, these AI-based frameworks suggest a path toward semi-automated or closed-loop PD management.

The PD9™ algorithm utilized in this study contributes to this evolving landscape by providing continuous, minute-by-minute assessments of motor symptom severity of bradykinesia and dyskinesia on a single, unified scale. This unique feature set (high temporal resolution combined with a single easy to understand scale) allows for precise characterization of symptom patterns and LD effects in naturalistic settings. Our findings emphasize the variability in LD response between individuals and across motor states, reinforcing the need for adaptive treatment paradigms supported by continuous, objective symptom monitoring.

This study has several limitations. The design was observational, without real-time dose adjustments based on sensor feedback. External confounding factors such as concomitant medications, different LD formulations (e.g., extended release), diet, sleep, or stress were not explicitly accounted for in this study. Additionally, while the PD9^TM^ algorithm has been validated against clinical rating scales [35,36], broader comparative studies with other systems and in larger populations are warranted.

## 5. Conclusions

This study highlights the critical role of continuous, algorithm-driven monitoring in addressing the challenges of individualized PD care. The PD9™ algorithm, through its ability to capture motor state fluctuations in real time, provides actionable insights into the impact of levodopa administration on symptom severity. Our findings revealed the necessity of optimizing the timing of medication intake on an individual level to maximize sustained symptom relief. They further suggest that motor-state-informed timing and dosage adjustments could enhance therapeutic efficacy while minimizing side effects. This approach not only supports more personalized treatment adjustments but also represents a step toward closing the loop in PD management. Future work should focus on integrating these tools into clinical workflows and exploring the relationship between motor states and LD pharmacokinetics through simultaneous biomarker analysis.

The combination of wearable technology and machine learning holds the promise to transform care for patients with Parkinson’s Disease, enabling more effective and precise interventions.

## Figures and Tables

**Table 1:** Clinical characteristics of the included patients. Categorical values are summarized by counts and percentages (%), numerical values by mean and standard deviation (SD) as well as median, minimum (Min) and maximum (Max).

	Overall (*n* = 67)
Gender	
Male	37 (55.2%)
Female	30 (44.8%)
Age	
Mean (SD)	67.0 (10.4)
Median [Min, Max]	68.2 [35.9, 86.0]
BMI	
Mean (SD)	25.3 (4.32)
Median [Min, Max]	24.8 [17.0, 36.1]
Disease duration	
Mean (SD)	9.09 (6.13)
Median [Min, Max]	9.00 [0, 26.0]
Hoehn & Yahr Stage	
1	3 (4.5%)
2	20 (29.9%)
3	29 (43.3%)
4	14 (20.9%)
5	1 (1.5%)
LED	
Mean (SD)	1070 (554)
Median [Min, Max]	1050 [100, 3750]

**Table 2:** Motor states experienced by patients during Levodopa intake.

Motor State	Count (%) (N = 218)
OFF	99 (45.4)
ON	68 (31.2)
Dyskinetic	51 (23.4)

## Data Availability

The data supporting the conclusions of this article will be made available by the authors on reasonable request.
